# Endothelial Mesenchymal Transition: Comparative Analysis of Different Induction Methods

**DOI:** 10.1186/s12575-016-0040-3

**Published:** 2016-04-27

**Authors:** Mariana T. Pinto, Dimas T. Covas, Simone Kashima, Claudia O. Rodrigues

**Affiliations:** Interdisciplinary Stem Cell Institute, University of Miami Leonard M. Miller School of Medicine, Biomedical Research Building, 1501 NW 10th Avenue, Room 826, Miami, FL 33136 USA; Instituto Nacional de Ciência e Tecnologia em Células-Tronco e Terapia Celular e Fundação Hemocentro de Ribeirão Preto, Ribeirão Preto, SP Brazil; Faculdade de Ciências Farmacêuticas de Ribeirão Preto, Universidade de São Paulo, Ribeirão Preto, SP Brazil; Faculdade de Medicina de Ribeirão Preto, Universidade de São Paulo, Ribeirão Preto, SP Brazil; Department of Molecular and Cellular Pharmacology, University of Miami Leonard M. Miller School of Medicine, Miami, FL 33136 USA

**Keywords:** Endothelial-mesenchymal transition, TGF-β, Hypoxia

## Abstract

**Background:**

Endothelial-Mesenchymal-Transition (EndMT) plays an essential role in cardiovascular development, and recently became an attractive therapeutic target based on evidence supporting its involvement in fibrosis and cancer. Important questions that remain to be answered are related to the molecular mechanisms that control EndMT in different organs and distinct pathological conditions. The lack of a detailed protocol for induction of EndMT and the assumption that TGF-β isoforms play similar roles on different types of endothelial cells, limit progress in the field. The aim of this study was to compare the induction of EndMT by TGF-β isoforms in endothelial cells of different sources, and define a detailed protocol for EndMT assessment in vitro.

**Results:**

We compared the dose-dependent effect of TGF-β isoforms, under normoxia and hypoxia, on the induction of EndMT in human coronary and pulmonary artery endothelial cells. Our results suggest that endothelial cells undergo spontaneous EndMT with time in culture under the conditions tested. The extent of EndMT induction by TGF-β was dependent on the dose and endothelial cell type. Furthermore, the potential of TGF-β to induce EndMT was reduced under hypoxia relative to normoxia.

**Conclusions:**

Our work suggests that the response of endothelial cells to TGF-β is intrinsic to the dose, cell type and environment. Optimization of induction conditions may be essential, as pathways triggering EndMT may vary during development and pathological conditions. Therefore, caution is needed regarding indiscriminate use of TGF-β to induce EndMT for mechanistic studies.

## Background

Endothelial-mesenchymal transition (EndMT) is a physiological process characterized by loss of cell-cell adhesion and cytoskeletal alterations, leading to changes in cell morphology and acquisition of invasive and migratory properties [1, 2]. During EndMT, the expression of the endothelial markers vascular endothelial cadherin (VE-cadherin), CD31 and von willebrand factor (vWF), has been reported to be reduced, and followed by an increase in the expression of the mesenchymal and smooth muscle markers vimentin, fibronectin, fibroblast-specific protein-1 (FSP-1), alpha-smooth muscle actin (α-SMA), SM22-α and calponin [1, 2]. EndMT plays an essential role in cardiovascular development [3], and has gained clinical relevance after discovery of its participation in fibrotic diseases of the kidney, heart and lung [4–9], and also during cancer progression [10, 11].

Mechanistic studies have demonstrated the involvement of different growth and pro-inflammatory factors on the induction of EndMT, although a special role has been assigned to members of the transforming growth factor-β (TGF-β) family [12–15]. Progress in the field has been limited by the lack of a detailed EndMT induction approach, the use of endothelial cells obtained from different species and tissues, and indiscriminate use of different isoforms TGF-β as potent inducers of EndMT with variable doses and time frames. An important question to be addressed is if similar EndMT induction mechanisms occur in different tissues, especially considering the diversity of endothelial cells in different organs [16] and dose-dependent role of members of the TGF-β family [17, 18]. The aim of the present work was to evaluate the induction of EndMT using a detailed protocol generated based on literature reports to test the effect of different doses of TGF-β in two distinct endothelial cell lines of human origin. In addition, we tested the effect of hypoxia on the induction of EndMT, as ischemia is an important trigger of tissue fibrosis, and has been shown to induce the expression of TGF-β [19–23].

## Methods

### Cells Line and Culture Conditions

Human coronary artery (HCAEC) and microvascular pulmonary artery (HMPAEC) endothelial cells, isolated from single donors, were purchased from Lonza and maintained according to manufacturer’s instruction in microvascular endothelial growth media (EGM-MV). All cells were used between passages 5–8 maximum, and maintained under 37 °C and 5 % CO_2_ humidified atmosphere.

### Induction of EndMT by TGF-β Treatment

Endothelial cells (2x10^5^ total) were plated in 60 mm collagen coated culture dishes in EGM-MV media. A day later, the culture media was changed to remove unbound/dead cells. Two days after initial plating, cells were harvested from one dish for protein analysis to determine the baseline levels of endothelial and smooth muscle markers prior to EndMT induction. All other culture dishes were treated separately with different doses (2 ng/mL, 5 ng/mL, and 10 ng/mL) of TGF-β1 or TGF-β2 (R&D Systems) every day, for a total period of 5 days. TGF-β treatment was performed without media change to avoid stimulation by other growth factors present in the culture media. At the end of 5 days, cells were harvested for protein analysis and compared to baseline. Untreated cells harvested at the end of 5 days were used as control.

### Induction of EndMT by Hypoxia Exposure

Endothelial cells were plated and treated essentially as described above for the TGF-β EndMT induction protocol. Two days after initial plating and collection of baseline lysates, cells were randomly divided in two groups and placed under hypoxia (1 % O_2_) and normoxia (room air condition at 21 % O_2_). In both cases, cells were maintained for a total period of 5 days without media change, except that they were also treated in parallel with TGF-β1 and TGF-β2 to determine the combined effects of hypoxia and TGF-β. At the end of 5 days, cells were harvested for protein analysis and compared to baseline.

### Evaluation of EndMT Induction

Induction of EndMT was assessed by Western Blot Analysis. Prior to lysate collection, cell viability was determined by microscopy under bright field and cell debris removed by 3 washes of monolayers with phosphate buffer saline. Cell lysates were prepared in RIPA buffer and protein concentration estimated using Bradford Assay (Bio-Rad) for normalization and loading of equal amounts of protein (30 μg total protein). SDS-PAGE and Western blots were performed according to standard procedures [24] using primary antibodies anti-CD31 (Cell Signaling Technology, #3528), anti-α-SMA (Sigma, #A2547), SM22-α (Abcam, #ab14106). Correct protein loading was confirmed by Ponceau Staining and probing with endogenous control antibodies anti-α-Tubulin (Santa Cruz, SC-58666) and anti-Gapdh (Cell Signaling, #2118). The same blot was probed with different antibodies without the need of stripping methods as proteins of interest differed in size. Chemiluminescent signal was detected using the ChemiDocTM XRS System and densitometry analysis performed using Quantity One software (Bio-Rad). The ratio between proteins of interest (CD31, α-SMA and SM22-α), and endogenous control (α-Tubulin or Gapdh) was calculated for data normalization. Graphs represent fold change calculated based on normalized data.

### Statistical Analysis

Results were analyzed for significance using One-Way ANOVA with Sigma-Plot and GraphPad Prism Software, followed by Holm-Sidak or Newman-Keuls multiple comparison post-tests. When applicable, ANOVA *on Ranks* was performed. Experiments were repeated 3 times for HCAECs and 4 times for HMPAECs. Differences between means were considered significant when *p* ≤ 0.05. All data are presented as means ± standard error.

## Results

### Effect of Culture Time on the Expression of Smooth Muscle Markers in Endothelial Cells

**Fig. 1:**
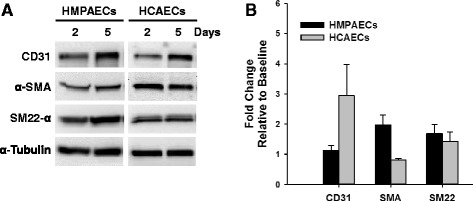
Time-dependent Change in the Expression of Endothelial and Smooth Muscle Markers in HMPAECs and HCAECs. **a** Representative Western blot image comparing changes in the expression of the endothelial marker CD31 and the smooth muscle markers α-SMA and SM22-α in HMPAECs and HCAECs with time in culture. **b** Quantification of endothelial and smooth muscle markers expression by densitometry analysis of western blots. Results were normalized to α-tubulin (loading control) and expressed as fold-change relative to baseline (day 5 versus day 2 after plating). CD31 (HMPAECs, *n* = 4; HCAECs, *n* = 3, non-significant); α-SMA (*n* = 3, non-significant), SM22-α (*n* = 3, non-significant)

The length of EndMT induction was variable among protocols described in the literature, without clear description of frequency in culture media replacement [7, 12, 13, 25], which can itself be a form of stress for any cell type. Therefore, we decided to start this study by investigating the expression of smooth muscle markers with time using commercially available human coronary artery (HCAECs) and microvascular pulmonary (HMPAECs) endothelial cells cultured for 5 days without media change. Western blot analysis showed that cultures of both cell types expressed detectable levels of the smooth muscle markers α-SMA and SM22-α as early as 2 days after plating (Fig. 1). After 5 days in culture, HMPAECs showed a trend towards increase in the expression of α-SMA and SM22-α by 1.98 ± 0.32 and 1.69 ± 0.30-fold, respectively, relative to baseline levels (2 days after plating). In HCAECs this increase was less pronounced for SM22-α (1.43 ± 0.31-fold) or slightly reduced in the case of α-SMA (0.81 ± 0.06-fold) (Fig. 1). Interestingly, the expression of the endothelial marker CD31 was increased in HCAECs by 2.94 ± 1.03-fold while it remained unchanged in HMPAECs (Fig. 1). Despite these trends, data quantification did not show any statistical significance (Fig. 1b).

### Effect of TGF-β on the Expression of Smooth Muscle Markers in Endothelial Cells

**Fig. 2:**
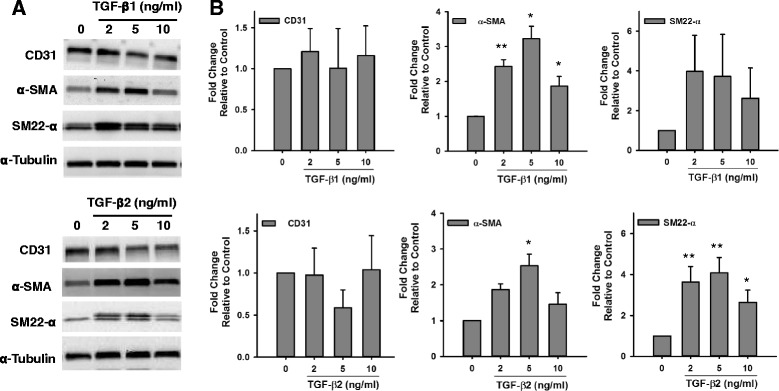
Effect of TGF-β on the Expression of Endothelial and Smooth Muscle Markers in HCAECs. **a** Representative Western blot images comparing changes in the expression of the endothelial marker CD31 and the smooth muscle marker α-SMA and SM22-α in HCAECs after exposure to different doses of TGF-β1 and TGF-β2. **b** Quantification of endothelial and smooth muscle marker expression by densitometry analysis of western blots. Results were normalized to α-Tubulin (loading control) and expressed as fold-change relative to control without TGF-β. (*n* = 3, *p < 0.05, **p < 0.005)

**Fig. 3:**
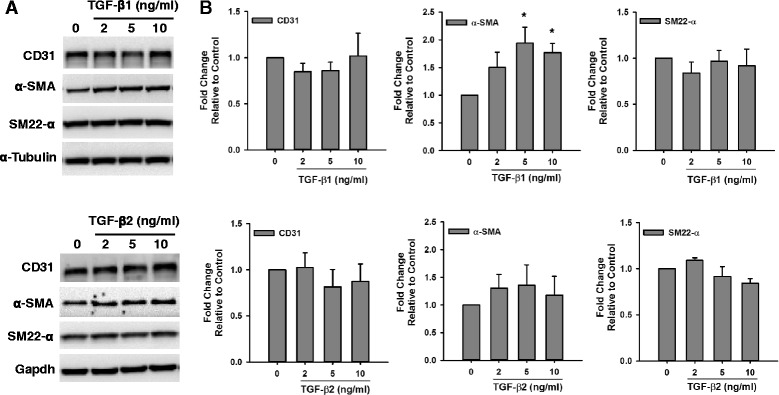
Effect of TGF-β on the Expression of Endothelial and Smooth Muscle Markers in HMPAECs. **a** Representative Western blot images comparing changes in the expression of the endothelial marker CD31 and the smooth muscle marker α-SMA and SM22-α in HMPAECs after exposure to different doses of TGF-β1 and TGF-β2. **b** Quantification of endothelial and smooth muscle marker expression by densitometry analysis of western blots. Results were normalized to α-Tubulin and Gapdh (loading control) and expressed as fold-change relative to control without TGF-β. (*n* = 3–4, *p < 0.05)

The involvement of TGF-β in EndMT has been widely described in the literature [12–15]. Nevertheless, a standard detailed protocol for its application as a potent inducer of EndMT in vitro has not been established. We tested the effect of different doses of TGF- β1 and TGF- β2 on the expression of smooth muscle markers in HMPAECs and HCAECs. We found that treatment of HCAECs with both TGF-β1 and TGF-β2 induced the expression of α-SMA and SM22-α (Fig. 2). Although all doses of TGF-β1 and TGF-β2 tested induced α-SMA expression, the one with highest effect was 5 ng/ml, which caused 3.23 ± 0.35 and 2.54 ± 0.32 -fold increase, respectively. Similarly, the expression of SM22-α was increased in HCAECs with all doses of TGF-β1 and TGF-β2 tested in a trend similar to α-SMA, though reaching higher fold changes at lower doses (3.97 ± 1.82 vs 2.43 ± 0.19 for 2 ng/ml TGF-β1, and 3.62 ± 0.76 vs 1.87 ± 0.16 for 2 ng/ml TGF-β2). The expression of endothelial markers is expected to decrease during transition. However, despite induction of smooth muscle markers in HCAECs with both types of TGF-β, only with 5 ng/ml TGF-β2 we observed a trend towards decrease in the expression of the endothelial marker CD31 to 0.59 ± 0.21-fold, although this decrease was not statistically significant. In HMPAECs only TGF-β1 showed a significant effect on the induction of α-SMA expression (Fig. 3), different from what we observed in HCAECs in which both types of TGF-β had an effect (Fig. 2). From the three TGF-β1 doses tested, 5 ng/ml and 10 ng/ml were the most effective, increasing α-SMA expression by 1.94 ± 0.29 and 1.77 ± 0.17-fold relative to untreated control, respectively. Interestingly, no significant changes were detected in the expression of SM22-α, like we observed in HCAECs. Furthermore, the degree of α-SMA induction in HCAECs was higher than in HMPAECs, 3.23 ± 0.35 vs. 1.94 ± 0.29 after treatment with 5 ng/ml TGF-β1.

### Effect of Hypoxia on the Expression of Smooth Muscle Markers in Endothelial Cells

**Fig. 4:**
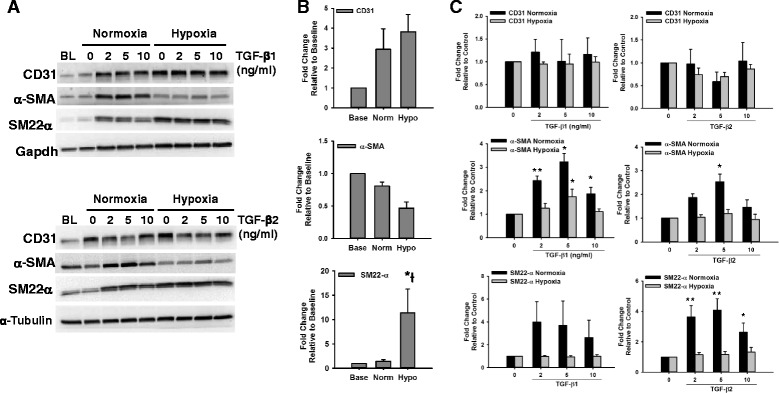
Effect of Hypoxia and TGF-β on the Expression of Endothelial and Smooth Muscle Markers in HCAECs. **a** Representative Western blot images comparing changes in the expression of the endothelial marker CD31 and the smooth muscle marker α-SMA and SM22-α in HCAECs after exposure to different doses of TGF-β1 and TGF-β2 under Normoxia (21 % O_2_) and Hypoxia (1 % O_2_). **b** Quantification of endothelial and smooth muscle marker expression by densitometry analysis of western blots comparing the effect of hypoxia to baseline and normoxia control. **c** Quantification of endothelial and smooth muscle marker expression by densitometry analysis of western blots comparing the effect of TGF-β1 and TGF-β2 under Normoxia (21 % O_2_) and Hypoxia (1 % O_2_). Results were normalized to α-Tubulin and Gapdh (loading control) and expressed as fold-change relative to baseline (BL) in B, and relative to control without TGF-β in C. (*n* = 3, *p < 0.05, **p < 0.005)

**Fig. 5:**
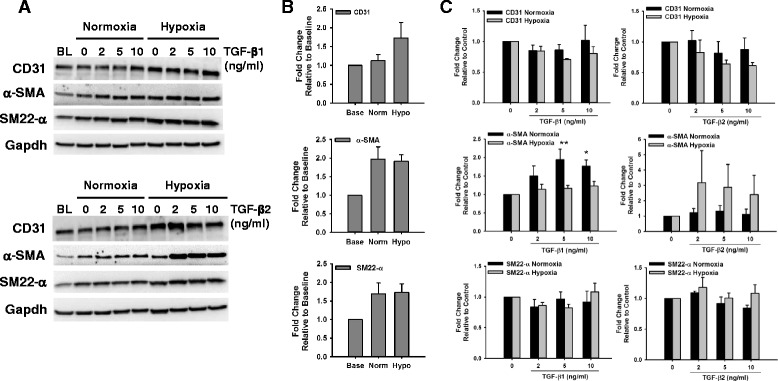
Effect of TGF-β on the Expression of Endothelial and Smooth Muscle Markers in HMPAECs. **a** Representative Western blot images comparing changes in the expression of the endothelial marker CD31 and the smooth muscle marker α-SMA and SM22-α in HMPAECs after exposure to different doses of TGF-β1 and TGF-β2 under Normoxia (21 % O_2_) and Hypoxia (1 % O_2_). **b** Quantification of endothelial and smooth muscle marker expression by densitometry analysis of western blots comparing the effect of hypoxia to baseline and normoxia control. **c** Quantification of endothelial and smooth muscle marker expression by densitometry analysis of western blots comparing the effect of TGF-β1 and TGF-β2 under Normoxia (21 % O_2_) and Hypoxia (1 % O_2_). Results were normalized to Gapdh (loading control) and expressed as fold-change relative to baseline (BL) in B, and relative to control without TGF-β in C. (*n* = 3–4, *p < 0.05)

Hypoxia has been known as an important inducer of tissue fibrosis in different pathological processes [23, 25–27], and its involvement in EndMT has been reported [23]. We tested the effect of 1 % hypoxia alone or in combination with TGF-β on the induction of smooth muscle markers relative to baseline (2 days after plating) and normoxia control (5 days later) in HCAECs and HMPAECs. In HCAECs, hypoxia exposure for 5 days caused a further decrease in the expression of α-SMA relative to control cells under normoxia from 0.81 ± 0.06- down to 0.47 ± 0.10-fold. Interestingly, the opposite was observed for SM22-α, which expression was significantly induced in HCAECs, relative to baseline, from 1.43 ± 0.31-fold in normoxia control to 11.41 ± 4.89 in hypoxia (Fig. 4a and b). When comparing both cell types, inverse changes were observed in the expression of α-SMA, and while it decreased in HCAECs (Fig. 4a and b), in HMPAECs it was increased up to 1.98 ± 0.32-fold (Fig. 5a and b). Induction of SM22-α by hypoxia was significant only in HCAECs (Fig. 4a and b). In HMPAECs, SM22-α expression was induced with time in culture without additional effect by hypoxia as observed in HCAECs. These results indicate that in HMPAECs, the induction of smooth muscle markers relative to baseline is independent of the oxygen levels and the marker tested, but related to the length of time in culture (Fig. 5a and b). Although the expression of α-SMA and SM22-α in HCAECs was induced by both TGF-β1 and TGF-β2 under normoxia, only TGF- β1 induced a significant increase in the expression of α-SMA under hypoxia of 1.75 ± 0.31-fold, without affecting the expression of SM22-α (Fig. 4c). Similar results were found for HMPAECs regarding the effect of TGF-β and α-SMA expression, without changes in SM22-α (Fig. 5). The endothelial marker CD31 was induced in both cell types under hypoxia, probably due to hypoxia-induced inflammatory response (Figs. 4 and 5).

## Discussion

The involvement and potential targeting of EndMT in fibrotic conditions has increased the research demand in the field. Most studies have pursued the use of TGF-β as the main tool for induction of EndMT based on its well-known contribution to endocardial cushion formation during development and fibrotic diseases [1, 26]. However, the lack of a detailed protocol that could be reproduced by the research community, the general assumption that all members of the TGF-β family play similar roles on the induction of EndMT, and that all endothelial cells respond to extracellular signals the same way is not realistic. In the present study, we investigated the effect of two members of the TGF-β family on the induction of EndMT using two different types of human adult endothelial cells. In addition, we tested the effect of hypoxia on the induction of EndMT, as ischemia is an important trigger of tissue fibrosis [27].

One of the first problems we encountered when starting our studies on EndMT was the lack of detailed and consistent information regarding the culture conditions, concentration and duration of TGF-β treatment. In addition, TGF-β treatment ranged in general from 2 to 10 days in serum-free conditions or media supplemented with serum [9, 12, 13, 28, 29]. Importantly, it was not clear if the culture media was replaced along treatment or if TGF-β was added without media change. These are relevant information as serum-free conditions may induce massive endothelial cell death as early as 24 h (personal unpublished observations), and different pathways would be stimulated by culture media change, especially if serum-supplemented. Based on these considerations and findings from the literature, we designed a protocol with commercially available HMPAECs and HCAECs consisting of 5 days TGF-β treatment, without culture media change, in which only TGF-β was added daily to the culture. When reviewing the literature we found a wide range in doses of different TGF-β isoforms tested, with many studies tending to the use the high dose of 10 ng/ml. In addition, when TGF-β isoforms have been tested together, the same dose has been used for the different isoforms, assuming that they would act in a similar fashion, compared to when tested alone. This generates concern, especially in the case of TGF-β, which has been reported to trigger different, sometimes opposite, effects depending on the dose used [18]. Our results show that low-to-intermediate doses of both TGF-β1 and TGF-β2, below 10 ng/ml, are sufficient to induce the expression of α-smooth muscle actin in both HCAECs and HPAECs. These findings are supported by reports showing that low concentrations ranging from 0.25–2 ng/ml were sufficient to induce EndMT [29–31]. One important feature of EndMT is loss of the endothelial phenotype, characterized by decreased expression of cell surface molecules. Interestingly, in our study, we were not able to see a robust decrease in the expression of the endothelial marker CD31, as previously reported. In fact, we saw a high degree of variability in the expression of CD31. Similar observations have been reported in aortic valve endothelial cells, which retained CD31 expression after TGF-β1 treatment [27], and further supported by another study showing that TGF-β caused only a slight decrease in the expression of CD31 in pancreatic microvascular cells [8]. Changes in the expression of endothelial markers may not occur all at once and may also be specific to individual proteins. Kokudo et al reported that although TGF-β2 treatment caused a decrease in the expression of claudin-5, the expression of VE-cadherin was unaffected, suggesting a marker-specific effect [12]. It is possible that our findings indicate an intermediate phenotype of EndMT, which has partially undergone transition [32], as loss of the endothelial phenotype during transition has been proposed to occur after completion of EndMT [8].

Hypoxia has been shown to be a potent inducer of epithelial-mesenchymal transition (EMT) in fibrotic disease [20, 33] and cancer [34], through mechanisms that may involve activation of TGF-β signaling [20, 21]. Similarly, the contribution of hypoxia to EndMT has also been demonstrated [19, 23, 35, 36]. In the present study we show different results regarding the expression of α-smooth muscle actin after exposure to hypoxia. We found that the expression of α-SMA in HMPAECs showed a tendency towards increase at endpoint under normoxia, relative to baseline, similar to what was observed under hypoxia. This result suggests that the increase in the expression of these proteins was probably related to the length of time they were maintained in culture along the experiment. Coexpression of smooth muscle markers in endothelial cells has been previously described [14, 29, 31, 37]. Freshly purified bovine adult arterial endothelial cells showed spontaneous and progressive increase in the expression of smooth muscle markers with time in culture, triggered shortly after cell isolation [31]. Paranya et al showed that certain clones of endothelial cells undergo EndMT independent of TGF-β treatment, when grown under low-serum conditions [27]. We may speculate that in our case, by not changing the culture media for a period of 5 days, nutrients are depleted, mimicking low-serum condition. Interestingly, in HCAECs exposed to hypoxia we found that α-SMA expression was decreased relative to baseline and normoxia control, different from recently published findings showing a significant increase in the expression of this marker after exposure to hypoxia [23]. The reason for these inverse findings on the expression of α-SMA in HCAECs is not clear. We found that the induction effect of TGF-β in HCAECs α-SMA expression is significantly reduced under hypoxia relative to normoxia. These results suggest that mechanisms involved in the control of TGF-β signaling under hypoxic stress may play a role in α-SMA decreased expression. One possibility is an additive effect of endogenous TGF-β produced under hypoxia and the amount supplemented, reaching high inhibitory levels.

## Conclusions

Optimization of EndMT induction conditions is critical, as pathways triggering this process may vary during development and pathological conditions. Our findings indicate that the response of endothelial cells to TGF-β is intrinsic to the dose, cell type and environment, suggesting that special attention is needed to the current design of EndMT studies and essential for reproducibility in future studies.
